# Supplementary material for: The impact of the genetic background on gene deletion phenotypes in Saccharomyces cerevisiae
Source: Mol Syst Biol. 2019 Dec 9;15(12):e8831. doi: 10.15252/msb.20198831 (PMC6901017; doi:10.15252/msb.20198831)
Supplement: Supplementary file 1 — Expanded View Figures PDF [file MSB-15-e8831-s001.pdf]

Expanded View Figures

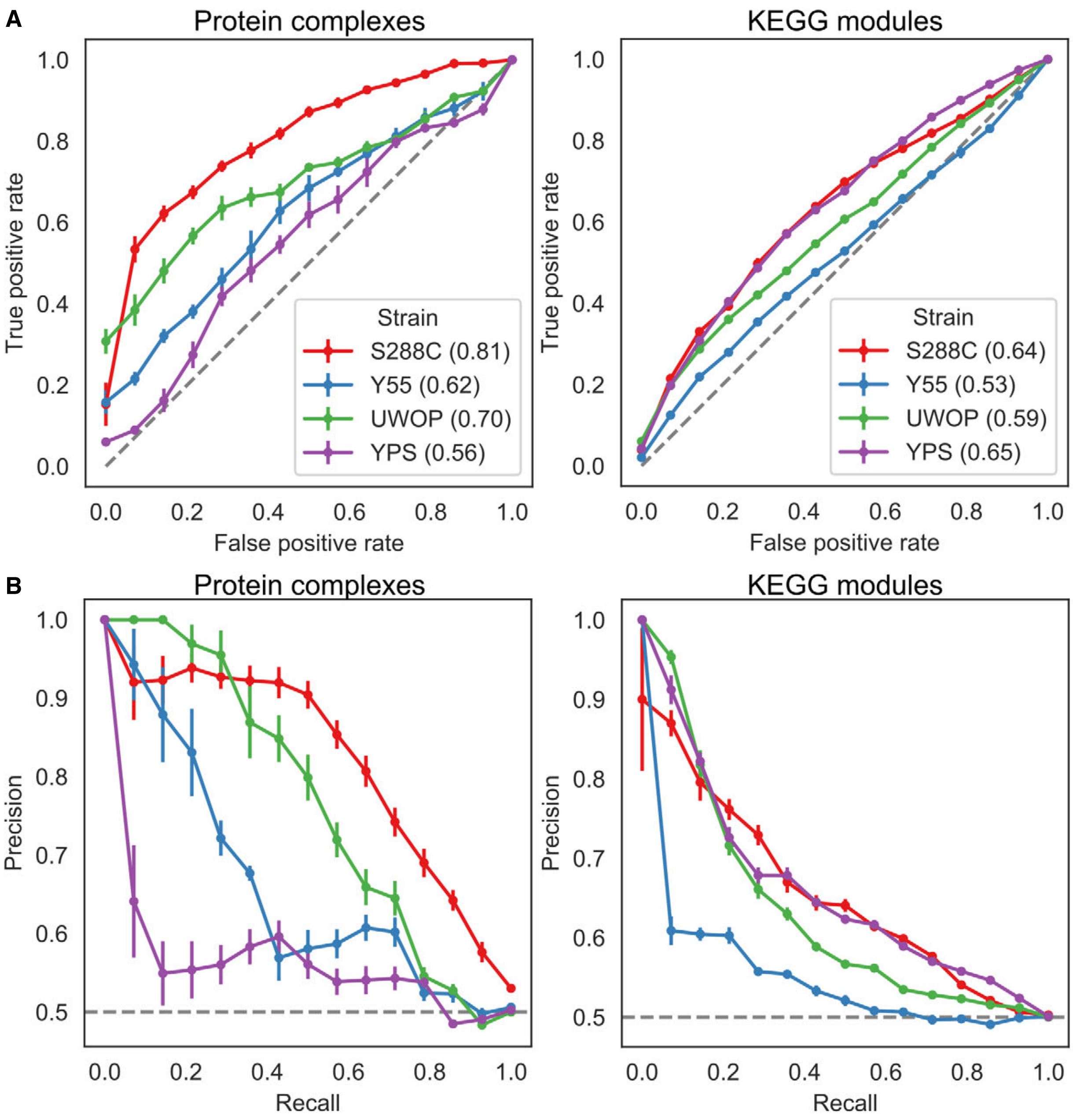

Figure EV1. Benchmarking of S-scores.

A, B Receiver operating characteristic (ROC) curves (A) and precision–recall curves (B) for two gene functional interaction sets across the four KO libraries. Numbers between parentheses in (A) represent the area under the curve (AUC). Error bars indicate the Mean Absolute Deviation over 10 replicates with random gene pairs as the negative set.

 © 2019 The Authors

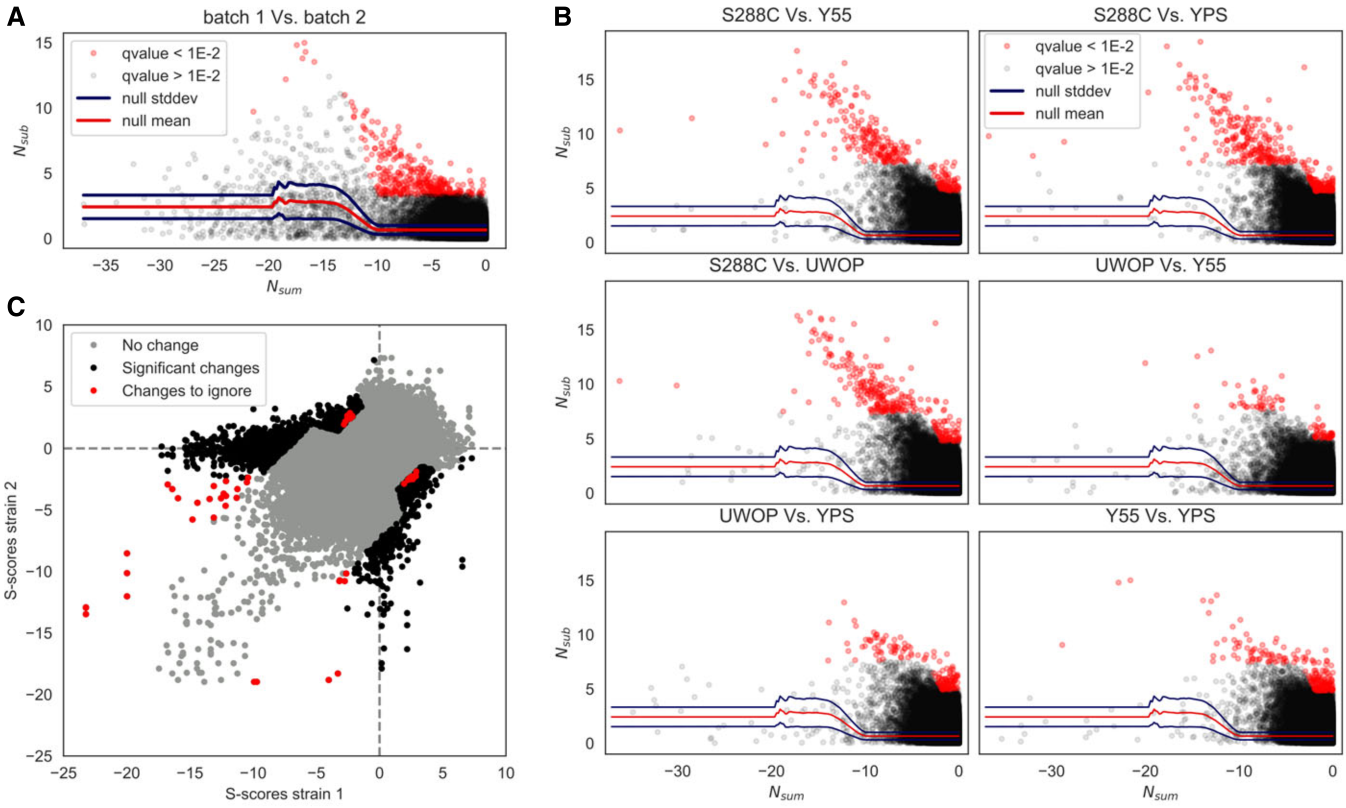

Figure EV2. Calling significant differences in S-scores.

A Null model to call significant S-score differences.

B All strains pairwise comparisons, indicating S-scores that change significantly between each pair.

C Overview of all strains' pairwise comparisons; "changes to ignore" refers to significant changes (corrected $P < 0.01$) that are inconsistent (see Materials and Methods).

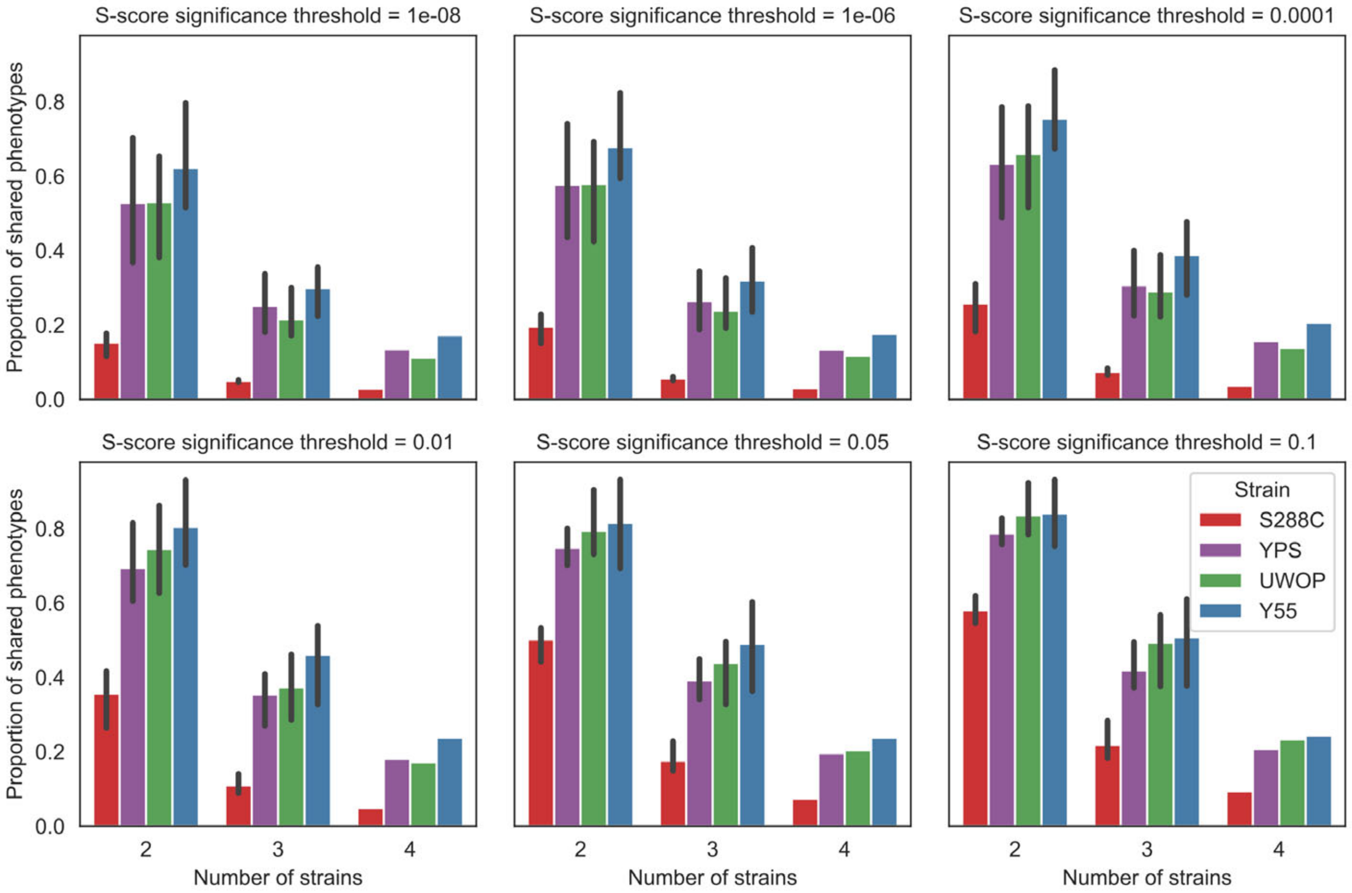

Figure EV3. General invariance of shared phenotypes when varying the significance threshold.

Proportion of shared phenotypes for different S-score significance thresholds on calling phenotypes. Error bars indicate the 95% confidence interval.

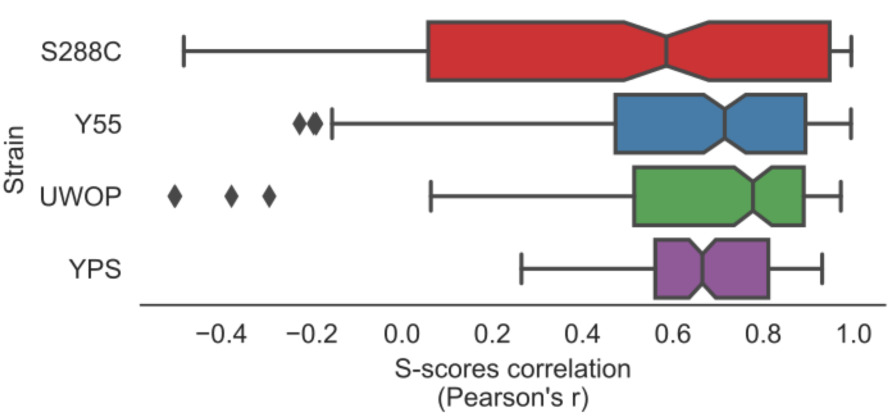

Figure EV4. Correlation between S-scores of the original KOs and the new ones.

Each data point corresponds to an individual gene. The central vertical line indicates the median, the box delimits the lower and upper quartile of the distribution and the whiskers extend to 1.5 times the inter-Quartile Range (IQR) plus the lower and upper quartile of the distribution, respectively.

© 2019 The Authors

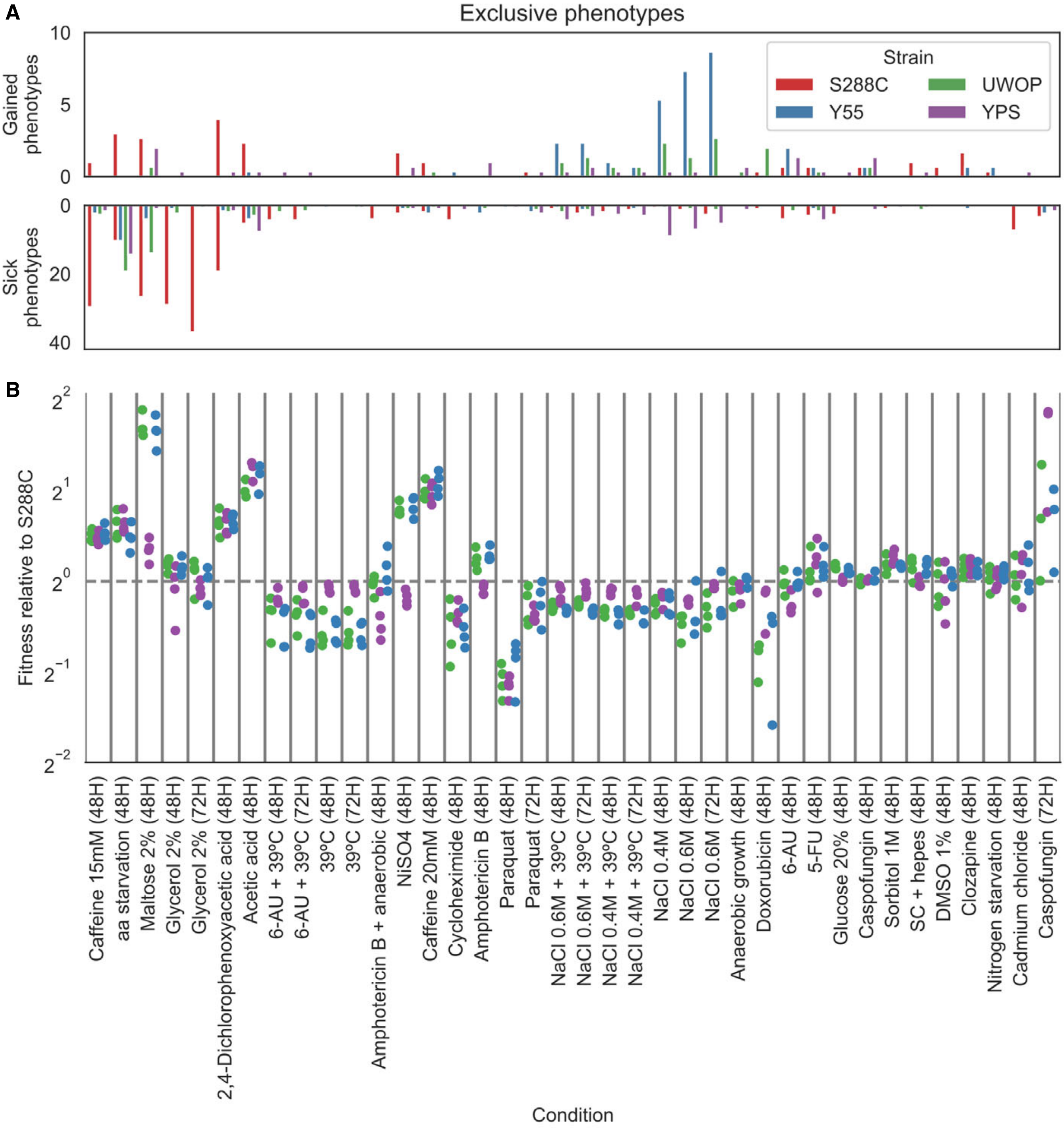

Figure EV5. Condition-specific changes in gene essentiality and fitness.

A Average number of KO phenotypes that are exclusive to a particular strain across all strains' pairwise comparisons. Conditions order is the same as panel (B).

B Wild-type fitness of each strain relative to S288C across all conditions; each dot represents a specific replicate where colony sizes were measured.

© 2019 The Authors

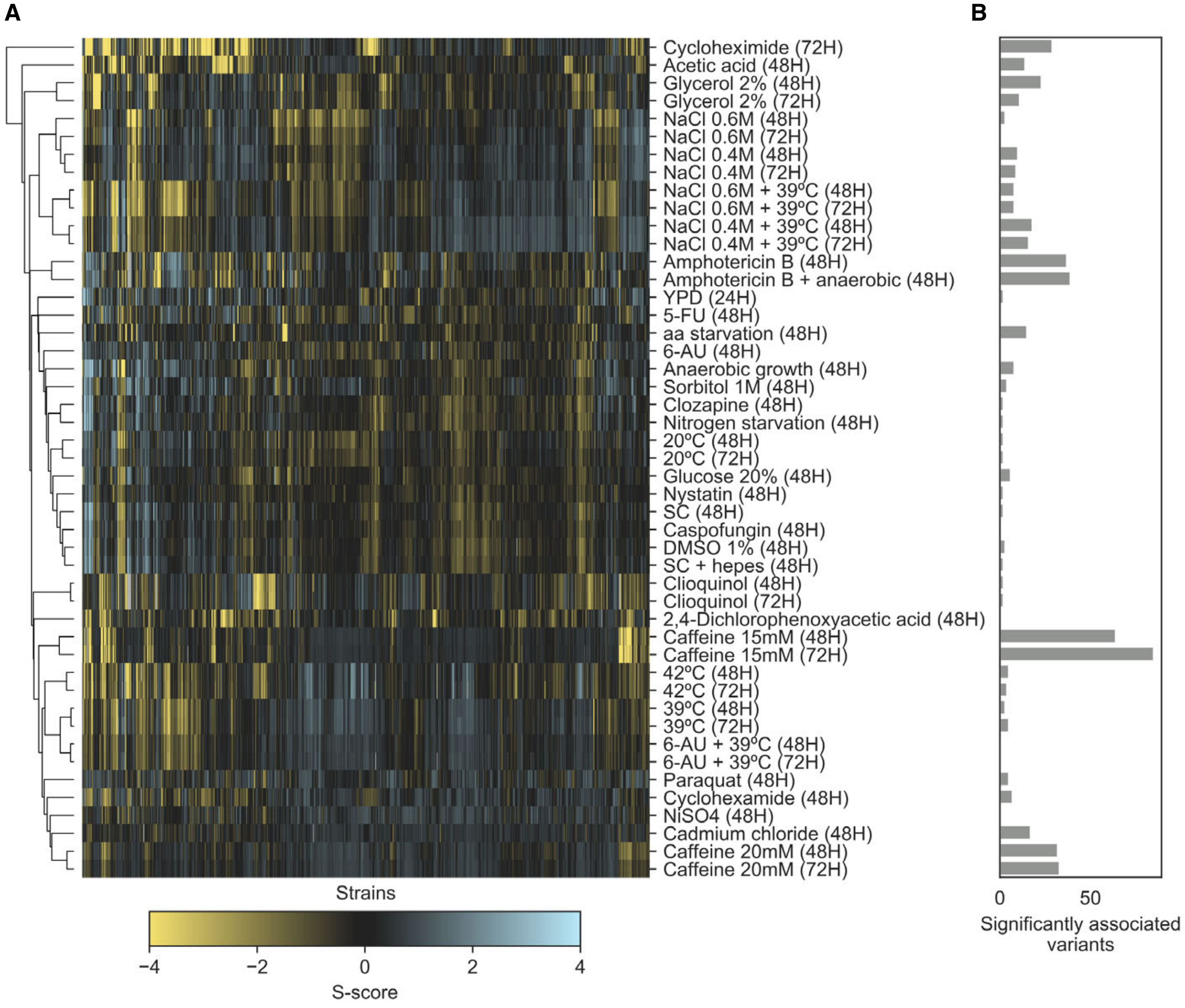

Figure EV6. Growth profiles of the 925 yeast natural isolates.

A Clustermap of S-scores for the whole set of conditions in which the natural isolates were tested in.

B Number of significantly ($P < 1E\text{-}6$) associated variants across the same conditions as in panel (A).

 © 2019 The Authors
