# Supplementary material for: The impact of the genetic background on gene deletion phenotypes in Saccharomyces cerevisiae
Source: Mol Syst Biol. 2019 Dec 9;15(12):e8831. doi: 10.15252/msb.20198831 (PMC6901017; doi:10.15252/msb.20198831)
Supplement: Supplementary file 9 — Table EV8 [file MSB-15-e8831-s009.zip › Table_EV8/Table_EV8/TableEV8_Legend.rtf]

Table EV8: differential expression of the Y55, YPS and UWOP strains compared to S288C in physiological conditions and in the presence of caffeine
